# The effect of vitamin D supplementation on the muscle damage after eccentric exercise in young men: a randomized, control trial

**DOI:** 10.1186/s12970-020-00386-1

**Published:** 2020-11-11

**Authors:** W. Pilch, B. Kita, A. Piotrowska, Ł. Tota, M. Maciejczyk, O. Czerwińska-Ledwig, E. Sadowska- Krepa, S. Kita, T. Pałka

**Affiliations:** 1 Institute for Basics Sciences, Faculty of Rehabilitation, University of Physical Education, Krakow, Poland; 2 Faculty of Physical Activity and Sport, University of Physical Education, Krakow, Poland; 3 Institute for Biomedical Sciences, Faculty of Physical Activity and Sport, University of Physical Education, Krakow, Poland; 4 Institute of Sport Sciences, The Jerzy Kukuczka Academy of Physical Education, Katowice, Poland

**Keywords:** Vitamin D, Eccentric exercise, Muscle cell damage, Il-1β, LDH, Myoglobin, CK

## Abstract

**Background:**

Vitamin D contributes to the optimal functioning of muscles. This study was designed to determine the modulating effect of vitamin D supplementation on the degree of muscle cell damage caused by eccentric exercise in young men.

**Methods:**

60 male volunteers (20–24 years old) taking part in this study were divided in two groups - with suboptimal (S) and optimal (O;) 25(OH)D plasma levels. These groups were randomly subdivided into groups with vitamin D supplementation (experimental: SE and OE) and controls (SC and OC). Before the supplementation (Test I) and after 3 months (Test II), participants were subjected to two rounds of eccentric exercise tests on a declined treadmill (running speed corresponded 60% VO2peak determined in each subject in incremental exercise test). During each test, blood samples used for determination of 25(OH)D, Il-1β, myoglobin (Mb) levels and CK, LDH activity were taken at three timepoints: before the test, 1 h and 24 h after it ended.

After distribution normality testing (Saphiro-Wilk test), statistical analyses were performed. Non-parametric: Kruskal-Wallis test and the Wilcoxon test were applied, and the Dunn-Bonferroni test as a post-hoc test.

**Results:**

In all groups, after 3 months, higher concentrations of 25(OH)D were indicated (SE *p* = 0.005; SC *p* = 0.018; OE *p =* 0.018; OC *p* = 0.028). SE and SC groups showed higher baseline concentrations of Il-1β and significantly higher concentrations of this interleukin after 1 h compared to groups with an optimal 25(OH)D level. After supplementation, the SE group reacted with a similar jump in concentration of Il-1β as the OC and OE groups. The change after 1 h after exercise in Test II was significantly different from that from Test I (*p* = 0.047) in SE group. Lower Mb concentrations indicated 1 h after exercise in Test II for SC and SE groups were indicated. CK activity did not differentiate the studied groups. Plasma calcium and phosphate disorders were also not indicated.

**Conclusions:**

The study has shown that vitamin D doses determined from the plasma concentration of 25(OH)D of individuals to match their specific needs can significantly reduce muscle cell damage induced by eccentric exercise.

## Introduction

Vitamin D has long been considered mainly a vital factor in the regulation of calcium-phosphate metabolism and an antirachitic agent. More recent reports indicate, however, that it may also have a role in pathomechanisms leading to the development of many illnesses, muscle atrophy and physical enfeeblement [1–4]. The dietary intake and cutaneous synthesis of vitamin D in individuals is measured by the blood concentration of its metabolite, 25(OH)D [5]. The importance of maintaining an optimal level of 25(OH)D is associated with the moderating effect of vitamin D on many physiological functions.

It is increasingly reported that vitamin D contributes to the optimal functioning of muscles in exercisers and non-exercisers, including older people who are physically inactive [6]. The optimal level of plasma 25(OH)D supports the muscle system during and after physical activity by helping maintain the proper levels of pro- and anti-inflammatory cytokines (mainly TNF-α, and interleukin-10) and thus suppressing inflammatory reactions [7–9]. Vitamin D enhances muscle cell protein synthesis by activating intracellular receptors, promotes physical capacity, muscle strength, mass, and endurance by maintaining the proper level of ATP, and reduces the Delayed Onset Muscle Soreness (DOMS). It is also reported to protect type-II (fast twitch) muscle fibres and shorten post-exercise recovery time [10]. On the other hand, vitamin D deficiency is indicated to impair motor coordination, muscle strength, and endurance, as well as increasing the risk of muscle damage. The values of the parameters improve as the blood 25(OH)D concentration returns to the optimal level [11, 12].

The most recent studies recommend vitamin D supplementation for patients with neuromuscular disorders [13, 14]. An association between low 25(OH)D levels and muscular enfeeblement, problems with climbing stairs and lifting objects, reduced grip strength, and muscular pain in seniors was found [15–17]. Histological examination of skeletal muscles has demonstrated a relationship between fast-twitch fibre atrophy and suboptimal 25(OH)D levels [16], which confirmed the importance of vitamin D for correct muscle function**.**

Eccentric exercise causes greater damage to muscle cells and more severe post-exercise ailments compared with concentric exercise [18, 19]. The damaged muscle structures (myofilaments and sarcomeres) secrete large amounts of compounds such as myoglobin (Mb), creatine kinase (CK), and lactate dehydrogenase (LDH) to the bloodstream. The blood concentrations of these compounds, as well as of myosin heavy chains (MHC), fat acid-binding protein (H-FABP), and troponin isoform I (sTnI), are widely used as indicators of skeletal muscle damage [19–22].

There are some studies in the literature showing the effect of vitamin D supplementation on the level of damage to muscle fibres under the influence of exercise [8, 23–27]. Efforts with a predominance of eccentric contractions have been studied very rarely [8, 25, 26]. In view of the above findings, a study was designed to determine the modulating effect of vitamin D supplementation on the degree of muscle cell damage caused by eccentric exercise in young men.

## Material and methods

### Participants

Eighty young male volunteers were screened for the study. Eighteen of them were excluded for meeting the exclusion, criteria, i.e. the use of tobacco, alcohol, medications or dietary supplements within 4 weeks before study. Another 2 were excluded for extremely low levels of plasma 25(OH)D revealed by the biochemical analysis. The outcomes of interviews and preliminary physical examinations showed that all 60 men who were enrolled in the study were healthy individuals and had skin phototypes I-III [28], without metabolic disorders, and capable of performing exercise tests. Based on the short International Physical Activity Questionnaire (IPAQ) [29], their physical activity before the study was assessed as moderate or low (1385 ± 116 MET-min/week, on average). The diet of the participants was diverse and met the nutritional norms recommended by Poland’s National Food and Nutrition Institute [30].

### Study design

**Fig. 1:**
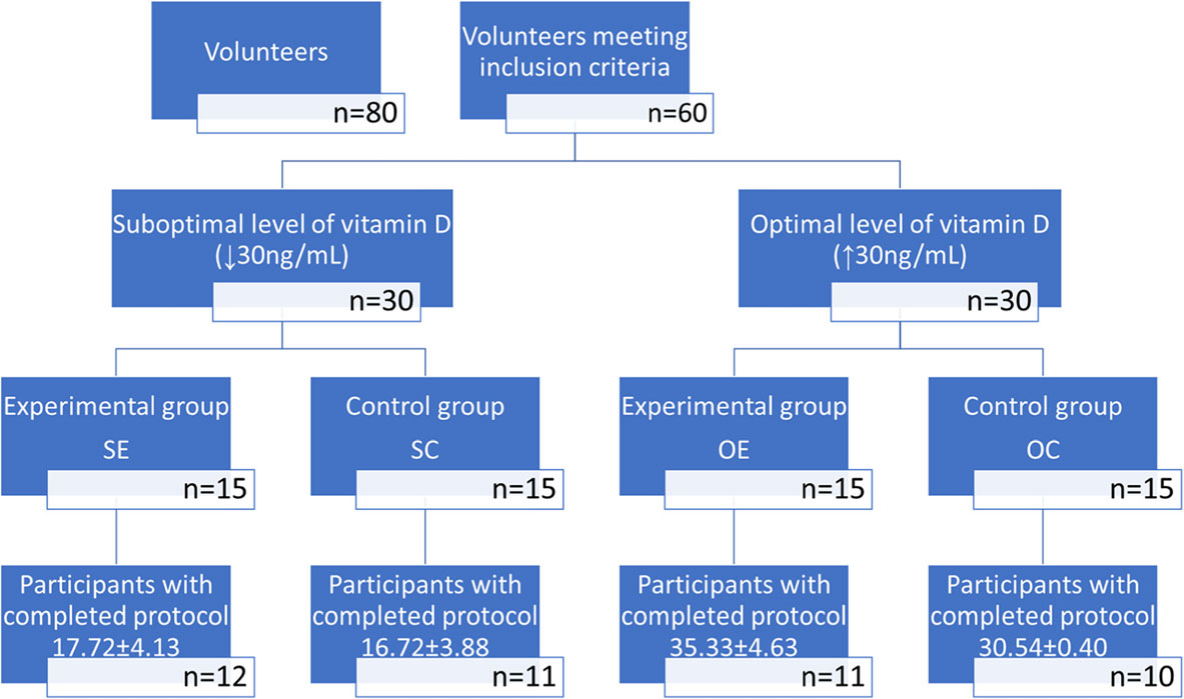
Patients diagram flew. Legend: SE-Experimental group with suboptimal level of vitamin D, SC-Control group with suboptimal level of vitamin D, OE-Experimental group with optimal lever of vitamin D, OC-Control group with optimal level of vitamin D. Plasma levels of vitamin D are shown as means ±SEM in ng/mL

The study began with the determination of 25(OH)D concentrations in the participants, which were used to divide them into two groups: S (*n* = 30) with suboptimal 25(OH)D levels (< 30 ng/ml; a mean of 17.05 ± 4.13) and O (*n =* 30) with 25(OH)D levels in the lower reference range (> 30 ng/ml; a mean of 33.33 ± 2.22). Groups S and O were then randomly subdivided into groups SE and OE (to be supplemented with vitamin D) and control groups SC and OC (placebo) (Fig. 1).

Before venous blood samples were taken for biochemical tests, participants underwent basic anthropometric measurements and were assessed for body mass and body composition (InBody 220 Biospace, Korea). The results of the measurements are presented in Table 1.

**Table 1:** Somatic characteristics of study participants

Group	BH [cm]	BM [kg]	BMI [kg/m^**2**^]	LBM [kg]	F%	FAT [kg]
$\overline{\boldsymbol{x}}$	180.20 ± 5.96	78.80 ± 8.34	24.26 ± 2.58	66.39 ± 6.31	12.41 ± 4.64	16.16 ± 4.08
**SE**	177.30 ± 5.03	77.99 ± 10.42	24.1 ± 3.07	64.68 ± 7.14	13.31 ± 4.49	16.82 ± 3.89
**SC**	182.86 ± 7.01	76.72 ± 7.41	23.03 ± 2.80	64.79 ± 6.43	11.93 ± 5.52	15.30 ± 6.20
**OE**	180.71 ± 5.94	75.26 ± 6.11	23.80 ± 1.58	65.54 ± 5.08	9.72 ± 4.83	15.51 ± 3.42
**OC**	181.33 ± 5.50	86.72 ± 3.55	26.47 ± 1.05	72.12 ± 2.95	14.60 ± 2.46	16.80 ± 2.53
***p***	0.6184	0.1409	0.1476	0.3606	0.1727	0.1367

*BMI* body mass index, *LBM* lean body mass, *F%* percentage of body fat, *FAT* body fat, *BH* body hight, *BM* body mass

Results are shown as means ± SEM (SE – experimental group with suboptimal level of vitamin D, SC – control group with suboptimal level of vitamin D, OE – experimental group with optimal lever of vitamin D, OC – control group with optimal level of vitamin D)

Peak oxygen uptake (VO_2_peak) was determined by having the participants perform incremental exercise tests. Its outcomes were used to set the running speed on a declined treadmill (eccentric exercise) so that it corresponded to 60% of VO_2_peak.

In the next step, participants were subjected to two rounds of eccentric exercise tests: before the supplementation with vitamin D or placebo (Test I) and after 3 months of supplementation (Test II). Blood samples were taken at three timepoints: before the test (baseline) and then 1 h and 24 h after it ended.

The study was conducted in the city of Krakow (50°03′41″N), Poland, between June and September 2017, at insolation of 800–850 W m^− 2^.

#### Exercise tests

On week before Test I, participants were subjected to an incremental exercise test to voluntary exhaustion on a conventional treadmill (Saturn 250/100 h, h/pCosmos, Germany) to determine their VO_2_peak. Heart rate (HR) was recorded in exercising participants using a cardiac monitor Polar 610S (Polar Electro, Finland).

##### Incremental exercise test

The purpose of the test was to establish participants’ VO_2_peak defined as the highest oxygen uptake recorded during the test. Its outcomes were used to set the intensity of eccentric exercise for Tests I and II. The test began with a 4- min warm-up during which participants ran at a speed of 8.0 km·h^− 1^ on a treadmill declined at 1^0^. After the warm-up, the running speed was increased by 1.0 km·h^− 1^ every 2 min. The test continued until voluntary exhaustion. The participants’ oxygen uptake during exercise was measured using an ergospirometer (MES, Poland).

##### Eccentric exercise test

The purpose of having the participants perform eccentric exercise was to induce muscle damage. During the test, the treadmill was inclined at − 10% and the speed was adjusted so that it corresponded to 60 ± 2% of VO_2_peak. To increase muscle cell damage, participants were loaded with rucksacks and metal weights accounting for 5% of their body mass with an accuracy of 0.1 kg [18]. Oxygen uptake was continuously monitored in participants by an ergospirometer to make sure that exercise intensity was equal to 60 ± 2% of VO_2_peak. The running speed was set for the test during the first 5 min of exercise (steady-state).

#### Supplementation

Groups SE and OE were supplemented with vitamin D (Vigantoletten 1000; Merck, Germany). Doses were calculated individually taking account of the participant’s body mass and baseline concentration of 25(OH)D using the formula proposed by Singh and Bonham [31].

Groups SC and OC received the placebo in the form of pills containing microcrystalline starch, which resembled in shape and colour vitamin D taken by groups SE and OE. All participants were instructed to take their pills once a day, after the main meal containing fats.

#### Biochemical analysis

Venous blood samples were taken from the participants using the BD vacutainer system (Becton Dickinson, USA) to tubes containing clot activator (for serum) or EDTA as the anticoagulant (for plasma) at seven timepoints: at baseline (to determine the participants’ initial 25(OH)D levels) and then before and 1 h and 24 h after each eccentric exercise test. Blood serum and plasma samples were obtained by centrifuging the collection tubes for 10 min at 2500 rpm in a MPW 350R laboratory centrifuge (MPW, Poland) and were frozen and stored until analysis at − 80 °C (Arctico ULF 390 PRC).

The plasma concentrations of 25(OH)D were analysed using the ELISA method (25(OH)D total, DRG, Germany, EIA-5396, assay sensitivity: 3.5 ng/ml, dynamic range: 3.5–130 ng/ml). With the immunoenzymatic method, the serum concentrations of myoglobin (Mb, DRG Germany, EIA-3955, assay sensitivity: 5 ng/ml, dynamic range 12–100 ng/ml) and of interleukin 1-β (IL-1ß, DRG, Germany, Easia-CE 14480, assay sensitivity: 0.35 pg/ml, dynamic range: 0–13.6 pg/ml) were determined. The activity of lactate dehydrogenase was measured by the colorimetric method (LDH, Assay Kit/Lactate Dehydrogenase Assay Kit, Colorimetric, Abcam, USA, ab102526, assay sensitivity: 1 mU/ml, dynamic range: 1–100 mU/ml). All tests were performed using a microplate reader Chromate 4300 (Awareness Technology, USA).

The activity of creatine kinase (CK) was determined using a Cobas C501 analyser (Roche Diagnostics, Switzerland) and the immunochemical method (dynamic range: 7–2300 mU/ml); the concentrations of inorganic phosphates (P) (dynamic range: 0.31–40.00 mg/dl) and the concentrations of calcium (Ca) (dynamic range: 0.80–150.00 mg/dl) were measured by the colorimetric methods; total protein concentrations (dynamic range: 0.20–36.00 g/dl) were assessed by the biuret method. When the test results were outside the dynamic range allowed by the method, the sample was diluted and tested again, and the outcome was converted as per the dilution ratio.

The values of all biochemical parameters obtained post-exercise were adjusted for the dehydration effect. To this end, the post-exercise plasma volume (%ΔPV) was calculated by applying formula [32] to the difference between the pre- and post-exercise volumes of total protein concentration, and then Kraemer’s and Brown’s formula was applied to adjust parameters’ values accordingly [33].

#### Statistical analysis

The results of statistical analysis (performed in Statistica 13) are presented as the means ± SEM. Variables were tested for distribution normality with the Shapiro-Wilk test. When the distributions were not normal, the non-parametric Kruskal-Wallis test and the Wilcoxon test were applied, and the Dunn-Bonferroni test as a post-hoc test. The level of significance (α) was set at *p* < 0.05.

## Results

Table 2 presents 25(OH)D concentrations in the study groups obtained before Tests I and II. The data show that the within-group differences before Test I were significant (*p* < 0.001). According to the post-hoc test results, groups SE and OE were statistically significantly different from OC (*p* = 0.05 and *p* = 0.028, respectively) and groups SC and OE from OC (*p* = 0.018 vs. *p =* 0.028).

**Table 2:** Plasma concentration of 25-OH-D [ng/ml] in study participants

Group	SE	SC	OE	OC	p (between groups)
**Test I**	19.10 ± 1.40	14.13 ± 5.08	30.53 ± 0.34	36.16 ± 4.47	0.000
**Test II**	41.23 ± 8.63	40.60 ± 10.62	41.24 ± 7.92	50.30 ± 8.49	0.127
**p (Test I vs. Test II)**	0.005	0.018	0.018	0.028	

Results are shown as means ± SEM (SE – experimental group with suboptimal level of vitamin D, SC – control group with suboptimal level of vitamin D, OE – experimental group with optimal lever of vitamin D, OC – control group with optimal level of vitamin D); p (between groups) Kruskal-Wallis test; p (Test I vs Test II) Wilcoxon test

In all groups 25(OH)D concentrations measured before Test II (*p* = 0.127) were higher than before Test I, but they did not significantly differentiate them (Table 2).

The baseline levels of interleukin 1β differed significantly between the groups. The highest level of this cytokine (*p* = 0.040) was found for the SE group. One hour after exercise, the OC group had significantly lower values of interleukin 1β than the other groups (*p* = 0.043). Post-hoc testing showed that interleukin 1β concentrations measured after 1 h eccentric exercise were significantly greater in the groups with suboptimal 25(OH)D levels (SC and SE) than in the other two groups, and that they decreased by hour 24. In three of the four study groups (SE, SC, and OE), changes in interleukin 1β concentrations brought about by Test II were statistically significant. In groups SE and OE (experimental), the concentrations of interleukin 1β measured 24 h after exercise were not significantly different from those measured after 1 h. The post-hoc test results showed that in the SC group the concentrations of interleukin 1β measured 1 h after exercise were statistically significantly lower than those obtained at hour 24.

Measurements performed after 3 months of supplementation with vitamin D did not show differences in either the pace or direction of changes in interleukin 1β concentrations in any of the groups (experimental and control) (Table 3). In almost all of them, the changes were statistically significant.

**Table 3:** Interleukin-1β concentration [pg/ml] in study participants

		SE	SC	OE	OC	p (between.groups)
**Test I**	1 h before	16.75 ± 16.50	9.80 ± 4.46	8.38 ± 6.14	4.00 ± 1.12	**0.040**
	1 h after	18.71 ± 17.77	13.58 ± 3.45	8.14 ± 5.93	5.33 ± 1.56	**0.007**
	24 h after	17.72 ± 17.51	6.59 ± 5.81	8.35 ± 7.15	4.76 ± 1.84	0.138
	p (inside.groups)	0.368	**0.002**	0.122	**0.050**	
**Test II**	1 h before	4.65 ± 4.60	8.42 ± 4.84	4.53 ± 4.02	3.43 ± 2.07	0.126
	1 h after	6.69 ± 5.12	12.66 ± 5.06	5.98 ± 4.80	5.59 ± 2.08	0.078
	24 h after	7.00 ± 4.04	8.10 ± 4.10	6.01 ± 3.77	5.35 ± 2.24	0.556
	p (inside groups)	**0.007**	**0.009**	**0.005**	0.066	
**p (Test I vs. Test II)**	1 h before	**0.047**	0.735	0.063	0.345	
	1 h after	0.386	0.612	0.398	0.463	
	24 h after	0.575	0.499	0.063	0.116	

Results are shown as means ± SEM (SE – experimental group with suboptimal level of vitamin D, SC – control group with suboptimal level of vitamin D, OE – experimental group with optimal lever of vitamin D, OC – control group with optimal level of vitamin D); p (between groups) Kruskal-Wallis test; p (inside groups) Friedman test for dependent means; p (Test I vs Test II) Wilcoxon test

A notable finding was a significantly lower concentration of interleukin 1β before test II in group OE, reflecting the effect of 3-month supplementation with vitamin D3 on the resting level of this protein.

An analysis of Mb concentrations obtained for Tests I and II did not find statistically significant differences between the groups. Regardless of the participants’ baseline levels of 25(OH)D, both the direction and time of changes in Mb concentrations were similar (Table 4).

**Table 4:** Changes of myoglobin [ng/ml] concentration in study participants sera

		SE	SC	OE	OC	p (between groups)
**Test I**	1 h before	26.13 ± 10.08	25.21 ± 6.48	18.65 ± 8.19	25.63 ± 8.22	0.295
	1 h after	143.0 ± 78.40	196.2 ± 111.3	126.6 ± 60.2	129.8 ± 51.41	0.544
	24 h after	34.45 ± 10.02	35.22 ± 20.06	31.95 ± 17.17	46.96 ± 18.48	0.122
	p (inside.groups)	**0.000**	**0.009**	**0.002**	**0.001**	
**Test II**	1 h before	25.52 ± 18.73	34.29 ± 12.32	45.24 ± 27.35	25.48 ± 11.02	0.180
	1 h after	71.65 ± 43.45	133.5 ± 53.66	75.33 ± 47.03	141.8 ± 85.32	0.099
	24 h after	45.91 ± 31.71	33.46 ± 17.71	21.42 ± 13.48	57.95 ± 51.27	0.874
	p (inside.groups)	**0.001**	**0.011**	**0.004**	**0.006**	
**p (Test I vs. Test II)**	1 h before	0.575	0,116	0.398	1.000	
	1 h after	**0.047**	**0,046**	0.091	0.612	
	24 h after	0.646	0,753	0.310	0.499	

Results are shown as means ± SEM (SE – experimental group with suboptimal level of vitamin D, SC – control group with suboptimal level of vitamin D, OE – experimental group with optimal lever of vitamin D, OC – control group with optimal level of vitamin D); p (between groups) Kruskal-Wallis test; p (inside groups) Friedman test for dependent means; p (Test I vs Test II) Wilcoxon test

Mb concentrations obtained 1 h after Test I were statistically higher in all groups, but those measured at hour 24 were significantly lower than at baseline. It was also found that in all groups Mb concentrations recorded 24 h after eccentric exercise were higher than at baseline. Similar changes were recorded for Test II (after 3 months of supplementation).

In the groups which had suboptimal 25(OH)D levels at baseline, Mb concentrations obtained 1 h after eccentric exercise were statistically significantly lower for Test II than for Test I. In the other groups, Mb concentrations measured at these two time points were not significantly different.

The between-group differences in LDH activity measured after Test I were not statistically significant. In all groups it was significantly higher 1 h after exercise and significantly lower at hour 24 (Table 5).

**Table 5:** Changes of Lactate dehydrogenase (LDH [mU/ml]) activity levels in plasma of study participants

		SE	SC	OE	OC	p (between groups)
**Test I**	1 h before	176.1 ± 31.37	175.8 ± 46.28	161.4 ± 20.59	156.0 ± 16.83	0.681
	1 h after	209.3 ± 27.52	228.0 ± 40.67	186.4 ± 22.70	181.9 ± 20.59	0.056
	24 h after	205.3 ± 19.60	197.8 ± 29.88	176.6 ± 19.7	174.6 ± 12.96	0.062
	p (inside.groups)	**0.050**	**0.006**	**0.001**	**0.012**	
**Test II**	1 h before	172.7 ± 24.40	219.8 ± 17.37	174.6 ± 24.51	162.9 ± 15.53	**0.006**
	1 h after	204.6 ± 22.45	249.5 ± 26.91	197.5 ± 21.80	188.6 ± 24.30	**0.018**
	24 h after	203.0 ± 18.54	215.2 ± 20.54	199.7 ± 33.56	186.7 ± 24.43	0.210
	p (inside.groups)	**0.004**	**0.006**	**0.003**	**0.015**	
**p (Test I vs. Test II)**	1 h before	0.672	0.075	0.066	0.612	
	1 h after	0.799	0.345	0.114	0.090	
	24 h after	0.499	0.249	0.111	0.107	

Results are shown as means ± SEM (SE – experimental group with suboptimal level of vitamin D, SC – control group with suboptimal level of vitamin D, OE – experimental group with optimal lever of vitamin D, OC – control group with optimal level of vitamin D); p (between groups) Kruskal-Wallis test; p (inside groups) Friedman test for dependent means; p (Test I vs Test II) Wilcoxon test

However, LDH activity measured 1 h before and 1 h after Test II was statistically significantly different between the groups. Post-hoc testing showed that in both cases it was higher in the SC group than in the other groups (*p* = 0.0004 and *p* = 0.015, respectively).

An analysis of LDH activity showed that it changed significantly in all groups after both tests (Table 5). One 1 h after exercise it was significantly higher, with considerably higher increases being noted for participants with suboptimal 25(OH)D levels than for the other ones (*p* = 0.003). In Test II, statistically significantly higher activity of LDH was also observed 1 h and 24 h after exercise.

The patterns of changes in LDH activity after Tests I and II were not statistically significantly different, showing that vitamin D supplementation did not have an effect on the levels of LDH activity after eccentric exercise.

An analysis of CK activity did not find significant between-group differences neither before Test I nor 1 h and 24 h later (Table 6). One hour after eccentric exercise the levels of CK activity were significantly higher than at baseline in all groups (*p* = 0.045), and still higher at hour 24 (*p* = 0.047).

**Table 6:** Changes of creatine kinase (CK[mU/ml]) activity in plasma of study participants

		SE	SC	OE	OC	p (between groups)
**Test I**	1 h before	274,1 ± 145,3	196,9 ± 84,34	216,7 ± 109,0	340,0 ± 246,2	0.454
	1 h after	363,1 ± 176,5	302,1 ± 101,2	273,0 ± 113,1	449,8 ± 270,3	0.496
	24 h after	785,3 ± 307,7	833,6 ± 437,3	669,4 ± 469,3	845,8 ± 290,3	0.475
	p (inside.groups)	**0.001**	**0.000**	**0.012**	**0.006**	
**Test II**	1 h before	283,4 ± 146,7	286,4 ± 160,8	207,6 ± 44,93	437,3 ± 144,7	0.056
	1 h after	366,0 ± 165,2	356,3 ± 171,2	272,7 ± 49,91	560,3 ± 209,0	0.055
	24 h after	1044 ± 606,0	900,0 ± 277,3	953,1 ± 626,8	971,2 ± 244,4	0.834
	p (inside.groups)	**0.001**	**0.000**	**0.001**	**0.002**	
**p (Test I vs. Test II)**	1 h before	1.000	0.093	0.735	0.463	
	1 h after	0.917	0.508	0.735	0.917	
	24 h after	0.237	0.721	0.612	0.345	

Results are shown as means ± SEM (SE – experimental group with suboptimal level of vitamin D, SC – control group with suboptimal level of vitamin D, OE – experimental group with optimal lever of vitamin D, OC – control group with optimal level of vitamin D); p (between groups) Kruskal-Wallis test; p (inside groups) Friedman test for dependent means; p (Test I vs Test II) Wilcoxon test

Similar changes were observed after Test II. CK activity measured 1 h after exercise was, again, significantly higher than at baseline in all groups, and its levels at hour 24 were higher than those obtained during two previous measurements.

Calcium and phosphate concentrations measured at the three time points were not significantly different (Table 7); they did not differentiate the supplemented groups from the non-supplemented ones, either.

**Table 7:** Changes in calcium (Ca [mg/dl]) and phosphates (P [mg/dl]) levels in blood plasma of study participants

Ca		SE	SC	OE	OC	p (between groups)
**Test I**	1 h before	2.51 ± 0.07	2.48 ± 0.14	2.45 ± 0.04	2.49 ± 0.07	0.274
**Test II**	1 h before	2.52 ± 0.08	2.46 ± 0.04	2.50 ± 0.05	2.47 ± 0.10	0.290
**p (Test I vs. Test II)**	1 h before	0.878	0.612	0.087	0.917	
**P**		**SE**	**SC**	**OE**	**OC**	**p (between groups)**
**Test I**	1 h before	1.17 ± 0.14	1.09 ± 0.26	1.10 ± 0.24	0.97 ± 0.50	0.927
**Test II**	1 h before	1.28 ± 0.15	1.30 ± 0.18	1.29 ± 0.25	1.24 ± 0.17	0.877
**p (Test I vs. Test II)**	1 h before	0.241	0.128	0.612	0.173	

Results are shown as means ± SEM (SE – experimental group with suboptimal level of vitamin D, SC – control group with suboptimal level of vitamin D, OE – experimental group with optimal lever of vitamin D, OC – control group with optimal level of vitamin D); p (between groups) Kruskal-Wallis test; p (inside groups) Friedman test for dependent means; p (Test I vs Test II) Wilcoxon test

## Discussion

The results of our study demonstrated that a 3-month supplementation with individually selected doses of vitamin D in the summer season is effective in restoring the optimal serum concentration of 25(OH)D. This outcome is consistent with the findings of other authors [23–25, 27, 34]. The supplemented participants’ concentration of 25(OH)D increased between baseline and end of month 3 by an average of 20–30 ng/ml. Increased 25(OH)D concentrations in the controls were probably due to the sunlight-induced *synthesis* of vitamin D. Furthermore, a 3-month supplementation with vitamin D of young, healthy men in the summer months in this study did not have a negative effect on their serum calcium and phosphate levels, indicating that it did not affect their calcium-phosphate metabolism.

The baseline differences in 25(OH)D concentrations were reflected in the values of muscle damage markers obtained after eccentric exercise. The post-exercise levels of IL-1β, CK, and Mb proved statistically significantly different from those recorded at baseline. The three markers are widely used to evaluate the effect of physical exercise on the human body, and their association with 25(OH)D concentrations has been reported before.

Sun X. et al. [9] have demonstrated that vitamin D can inhibit the production of pro-inflammatory cytokines (interleukin-17 (IL-17), interferon*-γ* (IFN-γ), and interleukine-6 (IL-6) in healthy adults. Studies with animal models have provided evidence that vitamin D can also reduce the production of IL-6, IFN-γ, and the tumour necrosis factor α (TNF-α). In view of the findings, we set out to investigate the possibility of muscle cell damage and inflammatory response to exercise, especially one involving mostly eccentric contractions, being less pronounced in persons with higher blood concentrations of 25(OH)D. Higher 25(OH)D levels are associated with lower creatine kinase, troponin I, and lactic acid dehydrogenase activity, and muscle soreness after training interventions [19].

To determine muscle cells damage caused by eccentric exercise, we measured the activity of CK and LDH and the concentration of Mb. In all participants the values of the markers were elevated post-exercise as a result of damaged muscle cells. The finding is consistent with the results reported by other authors [35, 36]. The participants with optimal 25(OH)D levels were found to have lower concentrations of pro-inflammatory metabolites IL-1β and Mb pre-exercise, which seems to indicate that vitamin D has a protective and anti-inflammatory effect. The finding is aligned with the results of studies pointing to the ability of vitamin D to inhibit the production of pro-inflammatory cytokines [9, 25]. The participants with optimal 25(OH)D levels also had lower concentrations of IL-1β and Mb post-exercise compared with those with suboptimal 25(OH)D levels, implying that vitamin D can protect muscle cells from the impact of high-intensity exercise. Several mechanisms have been reported that may be responsible for this beneficial effect in skeletal muscle [37]: genomic effects of vitamin D receptors (VDR) [38]; stimulation of oxygen uptake by heme-containing proteins co-working with cytochrome enzymes that could potentially affect the binding affinity of oxygen to hemoglobin [39].

The use of LDH activity as a measure of muscle cell damage was based on the observation that exercise boosts LDH activity proportionally to its intensity and duration. It was notable that in the participants with suboptimal 25(OH)D levels the post-exercise activity of LDH was significantly higher than in those who had optimal levels of this metabolite. A lower activity of CK measured 24 h after eccentric exercise in the latter suggested that vitamin D reduces exercise-induced damage to the cell membranes of skeletal muscles.

Similar findings have been reported from other studies. The authors of a study with rats subjected to intense exercise reported lower activity of CK and LDH and concentrations of IL-6 and TNF-α post-exercise in animals receiving vitamin D supplementation compared with unsupplemented controls [40]. The result led to conclude that vitamin D can play a key role in mitigating muscle damage and inflammation induced by exercise by modulating MAPK and NF-KB activation by VDR signalling.

In our study, LDH activity measured 1 h after Tests I and II was significantly higher in the SC group than in the other three groups, thus confirming the role of vitamin D as a factor in controlling and mitigating muscle cell damage caused by eccentric exercise. The effect was not observed to the same extent for Mb. In the OC and OE groups, the patterns of changes in Mb concentrations were significantly different. After 3 months of intervention, the plasma levels of 25(OH)D were higher in the SE groups (supplemented) and the SC groups (skin synthesis). In our opinion, this explains much smaller changes in Mb concentrations measured 1 h after exercise tests. More research and a larger group of participants are, however, needed to confirm the effect (false positive observations). The study by Żebrowska et al. [25] was aimed at investigating whether vitamin D supplementation can affect the relation between serum 25(OH)D concentrations and skeletal muscle biomarkers in professional athletes. A three-week low dosage vitamin D supplementation (2 × 1000 IU/day) caused an elevation of baseline serum 25(OH)D compared to baseline and to placebo group. The authors noticed that an increased 25(OH)D production seemed to have a significant effect on resting and eccentric exercise–induced skeletal biomarkers levels and proinflammatory cytokines, what had also been shown earlier by Barker et al. (2014). In professional athletes, higher 25(OH)D expression in response to vitamin D addition to the diet negatively correlated with biomarkers of skeletal muscle damage and that this effect was more pronounced during 24 h recovery period.

The number of papers showing a positive impact of vitamin D supplementation on optimizing athletic performance and recovery in intensely trained athletes is sparse [25, 41–43]. Except for our study, there are very few research papers evaluating the effects of vitamin D supplementation on muscle damage after eccentric exercise [8, 25]. Some authors suggested that different types of muscle contraction and/or different muscle groups may respond differently to vitamin D supplementation [17, 26]. But this hypothesis is still not confirmed.

Similar to our findings, Żebrowska concluded that 25(OH)D production after vitamin D supplementation has a significant effect on selected biomarkers of skeletal muscle damage and post-exercise proinflammatory cytokine levels [25]. A negative correlation was observed between vitamin D status and Mb concentration after supplementation.

One of the few works available on this topic indicates that that vitamin D supplementation attenuated the inflammatory biomarkers immediately following intensive exercise with both eccentric and concentric muscle contractions [8]. In Żebrowska’s study, with typical effort involving eccentric muscle contractions used in study protocol, lower post-exercise TNF-α levels and a tendency towards loweing IL-6 concentrations in a specifically trained supplementation group compared to the baseline levels was shown [25].

The level of 25(OH)D is influenced by a variety of factors of genetic (skin pigmentation (the phototype), the number of vitamin D receptors, etc.) and non-genetic origin (e.g. the duration of exposure to sunlight, the efficiency of intestinal absorption, age, fat tissue percentage, the use of vitamin D supplements, the geographical area, and the season) [44]. Changes in 25(OH)D concentrations recorded in our study after 3 months of intervention with vitamin D supplementation show that maintaining vitamin D homeostasis in countries characterised by seasonal variations in solar insolation and diet, such as Poland, is very difficult. In the study, significant increases in the plasma concentrations of 25(OH)D occurred in both the supplemented and the placebo group. However, the use of a formula with which the doses of vitamin D were individually selected to match participants’ needs allowed their plasma concentrations of 25(OH)D to be safely adjusted to the reference values despite the variable environment.

## Conclusion

The study has shown that vitamin D doses determined from the plasma concentration of 25(OH)D of individuals to match their specific needs can significantly reduce muscle cell damage induced by eccentric exercise. However, to be able to draw more definite conclusions about whether vitamin D supplementation influences the levels of muscle damage markers after eccentric exercise, a new diet-controlled study should be conducted in the autumn and winter months. It would also be instructive to analyse the patterns of changes in muscle damage markers in persons with skin phototypes other than phototypes I-III.

## Data Availability

The datasets used and/or analysed during the current study are available from the second author: Bartłomiej Kita (kitabart@yahoo.com) on reasonable request.
